# Ascorbic Acid: A New Player of Epigenetic Regulation in LPS-*gingivalis* Treated Human Periodontal Ligament Stem Cells

**DOI:** 10.1155/2021/6679708

**Published:** 2021-01-19

**Authors:** Guya D. Marconi, Luigia Fonticoli, Simone Guarnieri, Marcos F. X. B. Cavalcanti, Sara Franchi, Valentina Gatta, Oriana Trubiani, Jacopo Pizzicannella, Francesca Diomede

**Affiliations:** ^1^Department of Medical, Oral and Biotechnological Sciences, University “G. d'Annunzio” of Chieti-Pescara, 66100 Chieti, Italy; ^2^Department of Innovative Technologies in Medicine & Dentistry, University “G. d'Annunzio” of Chieti-Pescara, 66100 Chieti, Italy; ^3^Department of Neuroscience, Imaging and clinical Sciences-Center for Advanced Studies and Technology (CAST), University “G. d'Annunzio” of Chieti-Pescara, 66100 Chieti, Italy; ^4^Nove de Julho University, 01506-000 São Paulo, Brazil; ^5^Department of Psychological, Health and Territorial Sciences, School of Medicine and Health Sciences, “G. d'Annunzio” University, 66100 Chieti, Italy; ^6^“Ss. Annunziata” Hospital, ASL 02 Lanciano-Vasto-Chieti, 66100 Chieti, Italy

## Abstract

Periodontitis is usually sustained from microorganism of oral cavity, like *Porphyromonas gingivalis* (*P. gingivalis*). Periodontal disease is an infectious disease that afflicts a large number of people. Researches are investigating on the mesenchymal stem cells (MSCs) response to inflammatory events in combination with antioxidant substances. In particular, ascorbic acid (AA) increased cell proliferation, upregulated the cells pluripotency marker expression, provide a protection from inflammation, and induced the regeneration of periodontal ligament tissue. The purpose of the present research was to investigate the effects of AA in primary culture of human periodontal ligament stem cells (hPDLSCs) exposed to *P. gingivalis* lipopolysaccharide (LPS-G). The effect of AA on hPDLSCs exposed to LPS-G was determined through the cell proliferation assay. The molecules involved in the inflammatory pathway and epigenetic regulation have been identified using immunofluorescence and Western blot analyses. miR-210 level was quantified by qRT-PCR, and the ROS generation was finally studied. Cells co-treated with LPS-G and AA showed a restoration in terms of cell proliferation. The expression of NF*κ*B, MyD88, and p300 was upregulated in LPS-G exposed cells, while the expression was attenuated in the co-treatment with AA. DNMT1 expression is attenuated in the cells exposed to the inflammatory stimulus. The level of miR-210 was reduced in stimulated cells, while the expression was evident in the hPDLSCs co-treated with LPS-G and AA. In conclusion, the AA could enhance a protective effect in *in vitro* periodontitis model, downregulating the inflammatory pathway and ROS generation and modulating the miR-210 level.

## 1. Introduction

In the last few years, regenerative medicine based its success on the role of human mesenchymal stem cells (hMSCs). Human MSCs are characterized from two properties: self-renewal ability and multipotent differentiation potential [1]. Furthermore, hMSCs can differentiate and expand into mature cells as osteoblast, adipocytes, and chondroblasts and maintain the stem cell population [2]. They express specific surface markers, such as CD105, CD90, and CD73 and lack the expression for HLA-DR, CD45, CD34, and other hematopoietic markers [3]. These multipotent cells are located in different sites of the body. The bone marrow represents the gold standard tissue to isolate the hMSCs, but they can be also detected in the adipose and dental tissues, peripheral blood, umbilical cord blood, and amniotic membrane [4]. Several studies have been focused on alternative tissue sources for adult mesenchymal stem cells, like the dental pulp, dental follicle, apical papilla, periodontal ligament, and palatine tonsil [5]. In particular, hMSCs, derived from oral tissues, as dental pulp and periodontal ligament, are able to regulate tooth homeostasis and help tissue repair [6]. In particular, stem cells derived from human periodontal ligament (hPDLSCs) showed the ability to differentiate into mesengenic lineages. One of the most important features of hPDLSCs is the capacity to protect against infectious diseases related to their immunomodulatory properties [7, 8]. Periodontal disease is an infectious disease that leads to the progressive destruction of the periodontal ligament tissue and to the bone and tooth loss. Traditional clinical approaches for periodontal diseases are still insufficient to induce the full tissue regeneration of periodontium [9]. The hPDLSCs are not tumorigenic, maintain their classic phenotype and karyotype features in long-term passage cultures and are characterized by immunoregulatory properties [10, 11]. All hMSCs play an active role in the immune response; they interact with natural killer cells, dendritic cells, B lymphocytes, and T lymphocytes. Therefore, hMSCs avoid improper activation of T lymphocytes and limit the immune response during healing [12–14]. Nowadays, a great number of researches are investigating on the MSC response to inflammatory events in combination with antioxidant substances. Gram-negative bacteria showed on the outer of the cell membrane a component called lipopolysaccharide (LPS), and it is considered to be a major link for virulence in periodontitis [15]. Periodontitis is usually sustained from several microorganisms placed in the oral cavity, like *Porphyromonas gingivalis* (*P*. *gingivalis*, G), that afflicts a large number of people [16, 17]. Ascorbic acid (AA), known as vitamin C or ascorbate, consumed as a dietary supplement is a water-soluble vitamin that helps many enzyme activities [18]. AA is deeply involved in the reduction of reactive oxygen species (ROS) and showed a key role as an antisenescence agent [19]. When added to the cell culture, AA increased cell proliferation, upregulated the cell pluripotency marker expression [20], promoted MSCs regeneration of the periodontal ligament tissue, and protect inflammatory conditions induced by 2-hydroxyethyl methacrylate (HEMA) treatment reducing the intracellular inflammatory pathway [21].

Studies have shown that there exist a correlation between the periodontal diseases and some biomarkers, like microRNAs (miRNAs). miRNAs, noncoding small molecule RNAs, have influenced the development of periodontitis, as miR-146a, miR-128, and miR-200b [22–24]. miR-210 is related in the protection of the periodontitis; other than this, it has been identified in the triggering of several pathologies like cancers and immunological diseases [25]. Recent studies reported that miR-210 can be considered a key factor in the critical promotion of the osteogenic and angiogenic processes other than in cell survival [26, 27].

The purpose of the present study was to analyze the response of hPDLSCs to LPS-G, as a periodontitis *in vitro* model, alone or in presence of AA, in order to establish the potential protective role of AA on the inflammatory process triggered by LPS-G and in the preservation of the hPDLSCs reservoir.

## 2. Materials and Methods

### 2.1. Ethic Statement

Ethical Committee at the Medical School, “G. d'Annunzio” University, Chieti, Italy (number 266/April 17, 2014) approved the present study protocol. Informed consent form was filled and signed by all patients enrolled in the present research. All the procedures are in compliance with the 1964 Helsinki declaration and its later revisions, other than following the ethical standards of the institutional and/or national research committee.

### 2.2. Cell Culture Establishment

To collect hPDLSCs, we have enrolled six patients in good general health and without oral cavity diseases. They were to undergo surgical procedures to start the orthodontic treatment. After the collection, the periodontal ligament fragments were washed five times with phosphate-buffered saline solution (PBS, Lonza, Basel, Switzerland) [28]. The washed tissue fragments were placed in a culture dish in an incubator at 37°C in a humidified atmosphere of 5% CO_2_ in air. The mesenchymal stem cell growth medium-chemically defined (MSCGM-CD, Lonza) was used as a medium and changed every two days. Isolated cells were migrated spontaneously from the tissue fragments after two weeks of culture [29, 30].

### 2.3. Human PDLSC Characterization by FACS Analysis

Human PDLSCs were washed in PBS and then analyzed for CD14, CD34, CD45, CD73, CD90, and CD105 expressions [31]. Briefly, cells were stained for CD45, CD73, and CD90 with fluorescein isothiocyanate-conjugated antihuman antibodies and for CD14, CD34, and CD105 with phycoerythrin-conjugated antibodies. After staining procedures, FACStar-plus flow-cytometry system running CellQuest software (Becton-Dickinson, Mountain View, CA, USA) was used. All reagents used for flow cytometry were purchased from Becton Dickinson [32].

### 2.4. *In Vitro* hPDLSC Multilineage Differentiation

The capacity of hPDLSCs to differentiate into mesengenic lineages, as adipogenic and osteogenic differentiation, was evaluated by means of colorimetric detection and reverse transcription polymerase chain reaction (RT-PCR). In particular, hPDLSCs were placed in a 24 multiwell with a density of 2 × 10^4^ cells/well. To induce adipogenic differentiation, cells were maintained in MSCBM-CD supplemented with 10 mmol/L dexamethasone, 10 nmol/L 3-isobutyl-1-methylxanthine, 5 mg/mL insulin, and 60 mmol/L indomethacin for 28 days. The medium was refreshed every 3 days. Oil Red O solution (Sigma-Aldrich, Milan, Italy) was used to evaluate the adipogenic phenotype, staining the lipid droplet at cytoplasmic level. To induce the osteogenic commitment, the hPDLSCs were placed in a 24-multiwell with a density of 2 × 10^4^ cells/well. Cells were cultured with MSCBM-CD supplemented with 10 nmol/L dexamethasone, 10 nmol/L beta glycerophosphate (Sigma–Aldrich), and 50 mmol/L ascorbic acid for 21 days. Alizarin red S (Sigma-Aldrich) solution was used to stain the calcium depositions. Differentiated hPDLSCs were visualized to the inverted light microscopy Leica DMIL (Leica Microsystem, Milan, Italy). Furthermore, to evaluate the specific gene expression for adipogenic and osteogenic differentiation, RT-PCR has been performed. FABP4 and PPAR*γ* were evaluated for adipogenesis commitment, and RUNX-2 and ALP were analyzed for osteogenesis [33].

### 2.5. Study Design

All experiments were performed in triplicate with hPDLSCs at passage 2.

The study design is reported as follows:

- Untreated hPDLSCs, used as negative control (CTRL)
- hPDLSCs treated for 24 h with 50 *μ*g mL^−1^ with ascorbic acid (AA)
- hPDLSCs treated for 24 h with ultrapure lipopolysaccharide from *P*. *gingivalis* (tlrl-ppglps, InvivoGen, San Diego, CA, USA) 5 *μ*g mL^−1^ (LPS-G)
- hPDLSCs co-treated for 24 h with 50 *μ*g mL^−1^ with ascorbic acid (AA) and LPS-G 5 *μ*g mL^−1^ (LPS-G+AA)

### 2.6. MTT Assay

Cell viability of all experimental groups was determined using MTT colorimetric assay. Human PDLSCs were cultured in a 96-well at a density of 2 × 10^3^ cells/well. To evaluate the cell metabolic activity at 24, 48, and 72 h of culture, 20 *μ*L of MTT solution (CellTiter 96 AQueous One Solution reagent, Promega, Milan, Italy) was added to each well. Samples were maintained in the incubator, and after 3 h, the plates were read at 490 nm wavelength by means of a microplate reader (Synergy HT, BioTek Instruments, Winooski, VT, USA) [34].

### 2.7. Immunofluorescence and Confocal Laser Scanning Microscope (CLSM) Analyses

Sample fixation was performed with a solution of 4% of paraformaldehyde in 0.1 M of PBS (Lonza) [35, 36]. The following steps were performed: cells were permeabilized with 0.5% Triton X-100 in PBS for 10 min; samples blocking with 5% skimmed milk in PBS for 30 min [37]; primary antibodies (anti-NF*κ*B, 1 : 200, Santa Cruz Biotechnology; anti-MyD800, Thermo Fisher Scientific; anti-DNMT1, 1 : 200, EpiGentek; and anti-p300, 1 : 200, OriGene) incubation for 2 h at room temperature; and finally, secondary antibody (Alexa Fluor 568 red fluorescence-conjugated goat anti-rabbit antibody, 1 : 200, Molecular Probes, Invitrogen, Eugene, OR, USA) incubation for 1 h at 37°C. Cells were stained for 1 h with Alexa Fluor 488 phalloidin green fluorescent conjugate (1 : 400, Molecular Probes) and for 1 h with TOPRO (1 : 200, Molecular Probes) to mark the cytoskeleton actin and nuclei, respectively [38, 39]. The Zeiss LSM800 confocal system (Zeiss, Jena, Germany) has been used to acquire microphotographs.

### 2.8. Western Blot Analysis

Proteins (50 *μ*g) from all sample groups were processed as previously described [40]. Sheets were incubated 12 h at 4°C in with primary antibodies to NF*κ*B (1 : 500, Santa Cruz Biotechnology), MyD88 (1 : 1000, ThermoFisher Scientific), p300 (1 : 750, EpiGentek), DNMT1 (1 : 750, OriGene), and *β*-actin (1 : 1000, Santa Cruz Biotechnology) [41, 42]. Then, sheets were maintained at room temperature for 30 min with peroxidase-conjugated secondary antibody diluted 1 : 1000 in 1x TBS, 5% milk, and 0.05% Tween-20 [43]. The ECL method was used for band visualization, and the protein level were measured by means of the Bio-Rad Protein Assay (Bio-Rad Laboratories, Hercules, CA, USA) [44].

### 2.9. ROS Analysis

Human PDLSCs were seeded in 35 mm imaging dish (*μ*-Dish, ibidi GmbH, Gräfelfing, D). Cells were treated for 24 hours in culture medium containing 5 *μ*g mL^−1^ LPS-G; (hPDLSCs+LPS-G) or 5 *μ*g mL^−1^ LPS-G plus 50 *μ*g/mL ascorbic acid (hPDLSCs+LPS-G+AA) or 50 *μ*g/mL ascorbic acid (AA) or culture medium alone (control, hPDLSCs). At the selected time, the cells were washed with normal external solution (NES) containing (in mM): 125 NaCl, 5 KCl, 1 MgSO4, 1 KH_2_PO_4_, 5.5 glucose, 1 CaCl_2_, 20 HEPES, and pH 7.4 and incubated with 10 *μ*M of 2′,7′-dichlorodihydrofluorescein diacetate (H2DCFDA, Thermo Fisher Scientific) at 37°C in a humidified incubator (for 30 min) maintaining for all procedures the respective culture media treatments. At the end of dye incubation, the cells were washed with NES and observed in NES alone (hPDLSCs) or maintaining in NES plus hPDLSCs+LPS-G or AA alone. For each condition, confocal images were randomly acquired using a Zeiss LSM800 microscope (Carl Zeiss), equipped with an inverted microscope Axio-obserber.D1 and an objective W-Plan-Apo 40X/1.3 DIC. Excitation was fixed at 488 nm and emission was collected, setting the filter set over 505–530 nm. The acquisition settings were maintained constant between specimens. Fiji distribution of ImageJ software was used to analyze the captured images.

### 2.10. MicroRNA Quantization

MicroRNAs were extracted using the PureLink RNA mini kit (Life Technologies), treated with the RNase-Free DNase Set (Qiagen, Venlo, Netherlands) according to the manufacturer's instructions and quantified by means Nanodrop2000 (Thermo Scientific, Waltham, MA, USA). Gene sequences were from NCBI (http://www.ncbi.nlm.nih.gov), and RNA sequences for miR-210 were used into the Universal ProbeLibrary (UPL) Assay Design Center software (https://www.rocheappliedscience.com) to identify primers and UPL probe. Total RNA (50–200 ng) was retrotranscribed with High-Capacity cDNA Reverse-Transcription Kit (Life Technologies, Milan, Italy). MicroRNA quantization was performed using stem-loop RT primers designed with a modification to include the UPL #21 sequence-binding site [45]. The target amount, normalized to endogenous reference 18S/RNU44 and relative to a calibrator, was given by 2^-*ΔΔ*Ct^ and/or 2^-*ΔΔ*Ct^ methods (Life Technologies).

### 2.11. Statistical Analysis

Statistical evaluation has been performed using GraphPad 4.0 software using *t*-test and ordinary one-way ANOVA followed by post hoc Bonferroni's multiple comparisons tests. Values of *P* < 0.05 were considered statistically significant.

## 3. Results

### 3.1. Characterization of hPDLSCs Culture

**Figure 1:**
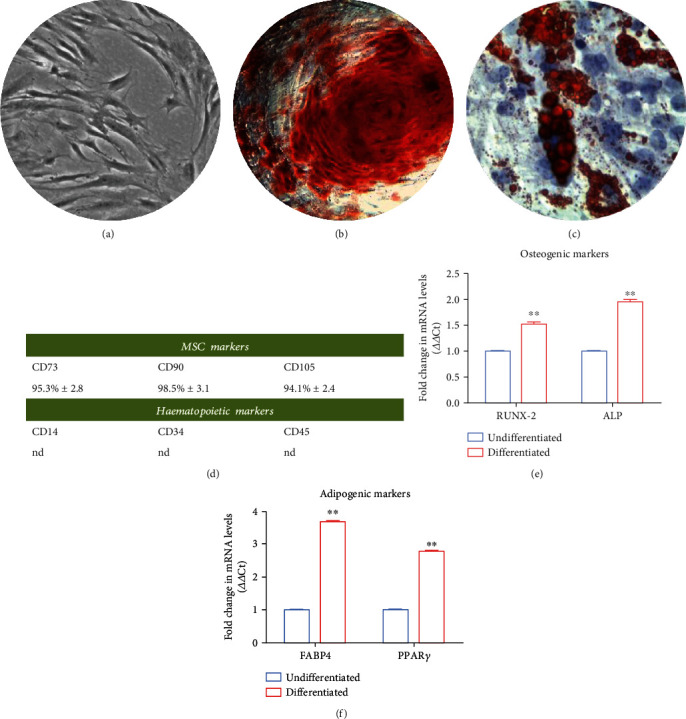
Phenotypic and functional characterization of hPDLSCs. (a) Representative images showing the morphology of hPDLSCs observed under light microscopy. (b) hPDLSC adipogenic differentiation: intracellular red lipid droplet stained with Oil Red O solution. (c) Human PDLSCs osteogenic differentiation: alizarin red staining of calcium deposition. (d) Cytofluorimetric analysis showed the positive expression for CD73, CD90 and CD105 and a negative expression for CD14, CD34, and CD45. (e) RT-PCR of RUNX-2 and ALP to detect the osteogenic differentiation. (f) RT-PCR of adipogenic markers. *P* < 0.001 was considered statistically significant (^∗∗^). Scale bar: 10 *μ*m.

Human hPDLSCs show a typical fibroblastoid morphology, and they are able to adhere to plastic surfaces (Figure 1(a)). The minimal criteria defined by the International Society for Cellular Therapy were used to characterize hPDLSCs. Human PDLSCs are able to differentiate into adipogenic and osteogenic phenotypes as demonstrated in Figure 1. Alizarin Red solution demonstrated with a red staining the calcium deposition (Figure 1(b)), and Oil Red O solution stained the intracellular lipid vacuoles (Figure 1(c)). Graph bars of RT-PCR validated the qualitative data, showing an upregulation of FABP4, PPAR*γ*, RUNX-2, and ALP in differentiated cells (Figures 1(e) and 1(f)). Flow cytometry results showed the positive expression for CD73, CD90, and CD105 and the negative expression for CD14, CD34, and CD45 (Figure 1(d)).

### 3.2. MTT Cell Viability Assay

**Figure 2:**
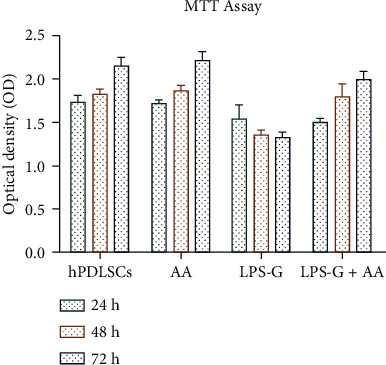
Cell viability analysis on hPDLSCs. Bar graph showed the cell viability of hPDLSCs treated with AA (50 *μ*g mL^−1^) and LPS-G (5 *μ*g mL^−1^) alone or in co-treatment with AA for 24, 48, and 72 h. The results are the mean (±S.E.M.) of three different experiments. ^∗∗^*P* < 0.001.

To evaluate the effects of AA, LPS-G, and AA in coadministration with LPS-G on hPDLSCs viable cells, the MTT assay was executed. During experiment, the hPDLSCs treated with AA showed a similar rate to the CTRL group, while hPDLSCs treated with LPS-G (5 *μ*g mL^−1^) evidenced a significant reduction of viable cells with respect to the other samples. After 24 h, the presence of AA reverts the effects of LPS-G showing a similar cell viability rate to the samples. A similar trend was reported after 48 and 72 hours of treatment with AA, LPS-G, and LPS-G+AA. The AA treatment showed beneficial effects, in terms of proliferation rate, after 24, 48, and 72 hours of treatment on both cell cultures (Figure 2).

### 3.3. Signaling Pathway NF*κ*B, MyD88, p300, and DNMT1 Analyses

**Figure 3:**
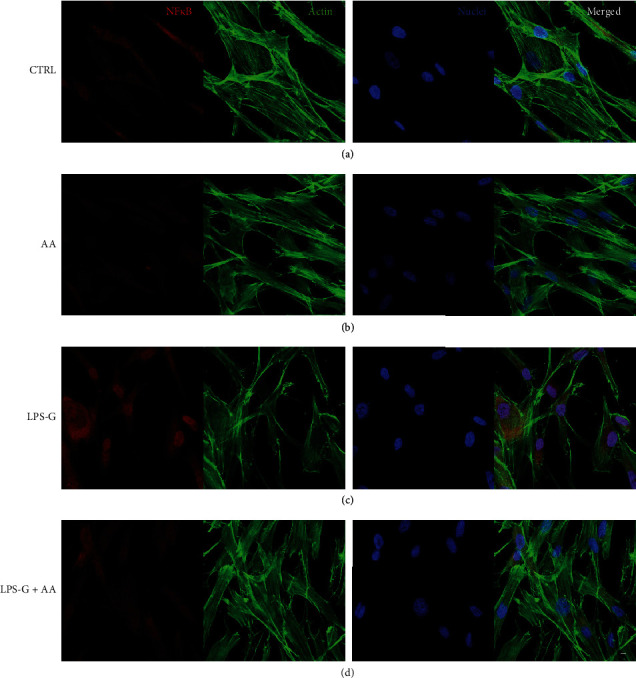
Immunofluorescence analyses of the expression of NF*κ*B. (a) Untreated hPDLSCs (CTRL). (b) Cells treated with AA (AA). (c) Cells treated with LPS-G (LPS-G). (d) Cells co-treated with LPS-G and AA (LPS-G+AA). NF*κ*B was stained in red fluorescence (Alexa Fluor 568 for secondary antibody). Cytoskeleton actin was stained in green fluorescence (Alexa-phalloidin 488). Cell nuclei were stained in blue fluorescence (TO-PRO). Merged image showed the overlap of all abovementioned channels. Scale bar: 10 *μ*m.

**Figure 4:**
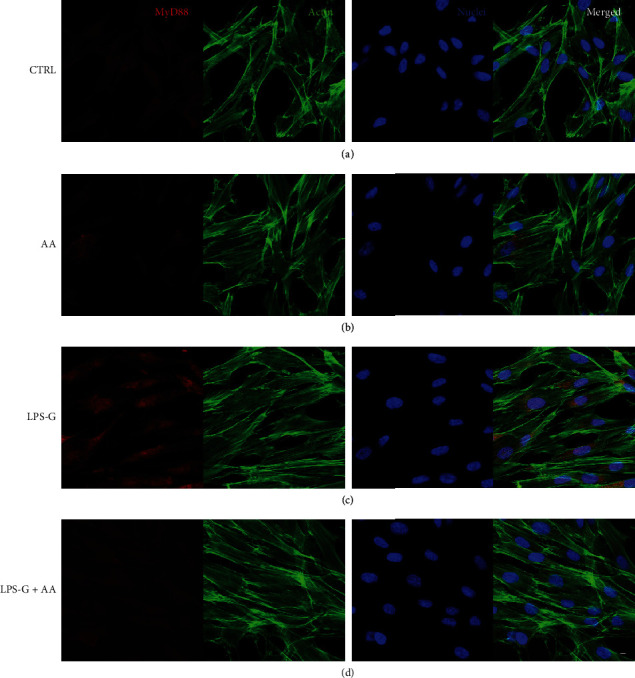
Immunofluorescence analyses of the expression of MyD88. (a) Untreated hPDLSCs (CTRL). (b) Cells treated with AA (AA). (c) Cells treated with LPS-G (LPS-G). (d) Cells co-treated with LPS-G and AA (LPS-G+AA). MyD88 was stained in red fluorescence (Alexa Fluor 568 for secondary antibody). Cytoskeleton actin was stained in green fluorescence (Alexa-phalloidin 488). Cell nuclei were stained in blue fluorescence (TO-PRO). Merged image showed the overlap of all abovementioned channels. Scale bar: 10 *μ*m.

**Figure 5:**
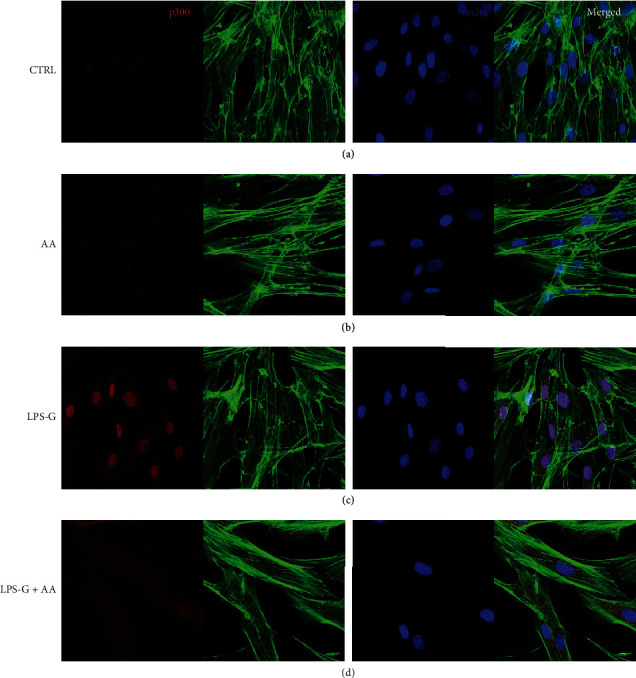
Immunofluorescence analyses of the expression of p300. (a) Untreated hPDLSCs (CTRL). (b) Cells treated with AA (AA). (c) Cells treated with LPS-G (LPS-G). (d) Cells co-treated with LPS-G and AA (LPS-G+AA). p300 was stained in red fluorescence (Alexa Fluor 568 for secondary antibody). Cytoskeleton actin was stained in green fluorescence (Alexa-phalloidin 488). Cell nuclei were stained in blue fluorescence (TO-PRO). Merged image showed the overlap of all abovementioned channels. Scale bar: 10 *μ*m.

**Figure 6:**
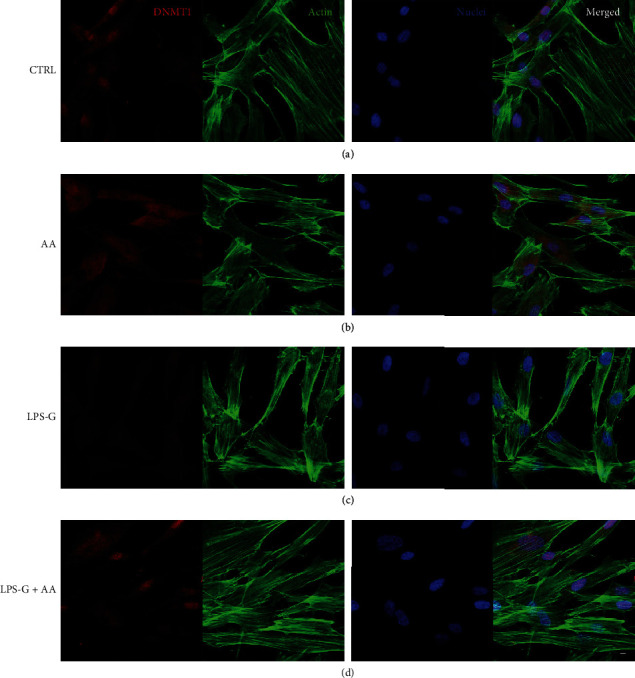
Immunofluorescence analyses of the expression of DNMT1. (a) Untreated hPDLSCs (CTRL). (b) Cells treated with AA (AA). (c) Cells treated with LPS-G (LPS-G). (d) Cells co-treated with LPS-G and AA (LPS-G+AA). DNMT1 was stained in red fluorescence (Alexa Fluor 568 for secondary antibody). Cytoskeleton actin was stained in green fluorescence (Alexa-phalloidin 488). Cell nuclei were stained in blue fluorescence (TO-PRO). Merged image showed the overlap of all abovementioned channels. Scale bar: 10 *μ*m.

Microphotographs captured by means of CLSM showed the intracellular expression of NF*κ*B, MyD88, p300, and DNMT1 (Figures 3–6). The hPDLSCs treated with LPS-G showed cell morphological changes compared to the untreated cells. The cells cotreated with LPS-G and AA evidenced a protective effect, hence the hPDLSCs a morphology reasonably comparable to the untreated hPDLSCs (CTRL). Furthermore, to confirm signaling network stimulated by LPS-G administration in hPDLSCs, the expression of NF*κ*B, MyD88, p300, and DNMT1 was examined after 24 h of culture. Immunofluorescence experiments to detect NF*κ*B, MyD88, p300, and DNMT1 localization were executed in hPDLSCs tested in all conditions stated above. Images in Figures 3–5 showed an increased fluorescence signal derived from NF*κ*B, MyD88, and p300 immunostaining in hPDLSCs treated with LPS-G compared to the hPDLSCs under other experimental conditions, in particular to the cells treated with AA. Immunofluorescence detection for DNMT1 showed a decrease in LPS-G-treated cells, meanwhile the expression level increases in cells cotreated with LPS-G and AA, similar to the CTRL and AA samples (Figure 6). LPS-G operates on the receptor and initiates a molecular cascade upregulating MyD88 and causing NF*κ*B to translocate at the nuclear level (Figures 4 and 5).

### 3.4. NF*κ*B, MyD88, p300, and DNMT1 Protein Expressions

**Figure 7:**
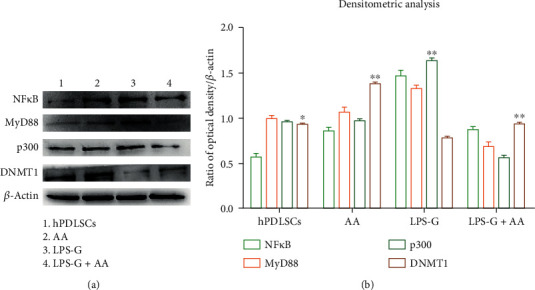
Protein expression. (a) Western blotting analysis of NF*κ*B, MyD88, p300, and DNMT1 expressions in hPDLSCs treated with LPS-G alone or in co-treatment of AA. (b) Densitometric analysis of protein bands expressed as a ratio of protein quantification normalized with *β*-actin. The error bars on these graphs evidence standard deviation (± SD). Graph bars showed the densitometric analysis. *β*-Actin was used as a housekeeping protein. The experiments were performed in triplicate. ^∗∗^*P* < 0.001, ^∗^*P* < 0.01.

Western blotting assay was evaluated to assess NF*κ*B, MyD88, p300, and DMNT1 protein expressions. In Figure 7, bands of NF*κ*B, MyD88, and p300 were augmented in LPS-G administered in hPDLSCs, while in cells treated with LPS-G+AA, a downregulation of NF*κ*B, MyD88, and p300 was existent. DNMT1 protein is expressed in hPDLSCs and in cells treated with AA and cotreated with LPS-G+AA.

### 3.5. ROS Production

**Figure 8:**
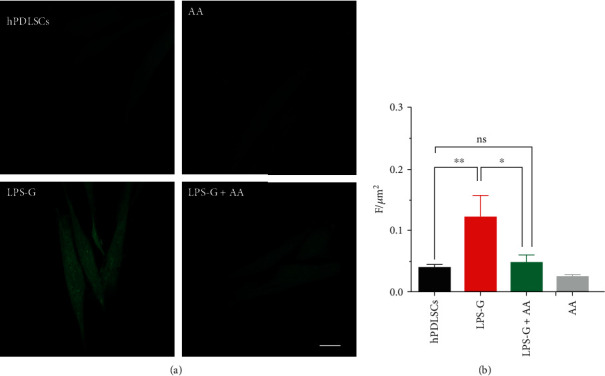
Single-cell ROS measurements. (a) Images of live cells loaded with H2DCFDA and acquired by confocal microscopy: hPDLSCs (control sample), LPS-G (LPS-G-treated cells), LPS-G+AA (LPS-G plus ascorbic acid-treated cells), and AA (ascorbic acid alone-treated cells). (b) Quantitative analysis of ROS production represented as arbitrary unit of fluorescence per cell surface unit (F/*μ*m^2^). Data are expressed as mean ± S.E.M (hPDLSCs *n* = 26, LPS-G *n* = 21, LPS-G+AA *n* = 16, AA *n* = 23; *N* = 3; ^∗∗^*P* < 0.001, ^∗^*P* < 0.01). Statistical analysis was performed by one-way ANOVA and post hoc Bonferroni). Scale bar = 20 *μ*m.

To verify the ROS production stimulated by LPS-G, hPDLSCs were loaded with the cell-permeant H2DCFDA a probe, used as an indicator for ROS. Figures were taken in live cells by means of confocal microscopy, and the single cell fluorescence recorded was finally offline investigated. In Figure 8(a), typical images acquired in our experimental circumstances are shown. Comparing the images taken, in the LPS-G-treated cells, there is an evident increase in emitted fluorescence with respect to the others, while the cotreatment of LPS-G in presence of AA appeared quite similar to that obtained in hPDLSC or AA alone. Quantitative results (Figure 8(b)) showed a growth in ROS production in 5 *μ*g mL^−1^ LPS-G-treated hPDLSCs vs. hPDLSCs (means ± S.E.M.: LPS-G 0.1 ± 0.02 vs. hPDLSCs 0.04 ± 0.005). The co-presence of ascorbic acid successfully appeared to be able to block the ROS production induced by LPS-G (LPS-G+AA 0.05 ± 0.001), while the AA alone did not modify the ROS basal levels acquired in hPDLSC samples (AA 0.040 ± 0.003).

### 3.6. miR-210 Expression

**Figure 9:**
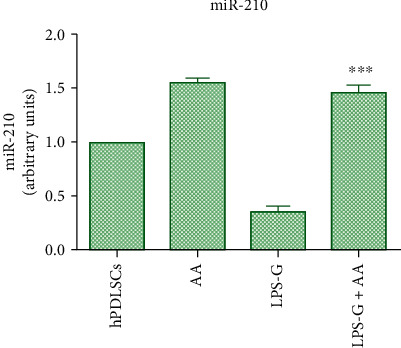
MicroRNA-210 expression. Graph bar showed the expression of miR-210 in all conditions. miR-210 was downregulated in cells treated with LPS-G, while in cells cotreated with LPS-G and AA was overexpressed. ^∗∗^*P* < 0.001.

miR-210 expression was downregulated in hPDLSCs administered with LPS-G, while in cells treated with AA and cotreated with LPS-G+AA, the miRNA-210 level is comparable to the CTRL group (Figure 9).

## 4. Discussion

Periodontitis is a chronic inflammatory illness, worldwide distributed and often correlated to many other chronic diseases such as cardiovascular disease, inflammatory bowel disease, rheumatoid arthritis, respiratory tract infection, and Alzheimer's disease, exhibiting a given attention in the relationship between oral and systemic health [3–5]. Periodontitis is sustained by a definite oral microorganism that is localized in the gum plaque, as *Porphyromonas gingivalis*, *Treponema denticola*, *Tannerella forsythia*, and *Aggregatibacter actinomycetemcomitans*. The periodontal disease worsening brings to the loss of periodontal ligament, alveolar bone, and consequently the tooth leakage [9].

The maintenance of the periodontal health becomes a real challenge to ameliorate the quality life of a wide part of population [10]. The human oral microbiome can be considered essential in the pathogenesis and in the development of the periodontal disease.

Periodontal tissue damage is a result of an aberrant host response to the bacterial infection and immune response activated from the polymorphonuclear leukocytes (PMNs) that are critically implicated versus the periodonto pathogens. This antimicrobial response at the infection site triggers numerous intracellular signaling pathways, including reactive oxygen species release (ROS), that can be considered the principal cause for the periodontal tissue injury in periodontal illness [46, 47].

The inflammation, involved in the stimulation and maintenance of the periodontitis, induced the epigenetic modifications in the periodontal ligament niche. Epigenetic modifications are a complex molecular mechanism with chemical modifications of DNA and their related proteins that lead to the activation or inactivation of some gene transcription [48].

In the present study, hPDLSCs were administered with LPS-G to mimic in vitro the periodontitis model.

The in vivo periodontitis scenario is very complex and involves the interaction of several cell bacteria and factors. The use of ultrapure preparation of LPS-G in a human cellular model could understand the mechanisms of the inflammatory response in periodontitis and the development of future clinical treatments. As previously reported, to study the periodontitis model, it is important to consider two key factors: (i) the LPS-G preparation, “standard” or “ultrapure” [49] and (ii) the in vitro model, mouse or human, to better reflect the in vivo situation. The use of mouse models could underestimate the role of LPS-G in the triggering and sustaining of human pathology [50].

LPS-G treatment activated the MyD88 and p300 signal transduction and induced NF*κ*B nuclear translocation. Meanwhile, the immunofluorescence and protein analyses showed a suppression in the expression of DNMT1 [51, 52]. As previously reported, DNMT1 expression is downregulated in oral epithelial cell and hPDLSCs treated with *P. gingivalis* (whole bacteria) or LPS-G [53].

AA supplemented with the diet exerts an antioxidant outcome, downregulating the free radical production and operating as a cofactor in cell functions [54, 55]. AA is also considered vital in the maintenance of periodontal health for its role in the downregulation of ROS production [56]. As earlier described, the role of AA in periodontal disease is in the avoiding and reducing the progression of the destruction process, stimulating the differentiation of periodontal ligament progenitor cells [57]. In the present study, the effects of AA in the periodontal disease in vitro model has been assessed in terms of cell viability, inflammatory pathways, gene expression, ROS production, and miRNA 210 expression. The treatment with AA increased the viability of hPDLSCs, while the LPS-G treatment showed a decrease in cell viability; moreover, the co-treatment of AA and LPS-G restore the conditions obtained in CTRL samples.

LPS-G treatment induces the inflammatory intracellular signaling cascades, as NF*κ*B, MyD88, and p300 pathways. NF*κ*B is a family of transcription factors implicated in the activation of inflammatory genes and in periodontal disease progression. NF*κ*B showed a redox-sensitive potential for ROS in different tissues, as endothelial and vascular smooth muscle; other than that, it has been extensively studied as a proinflammatory nuclear transcription factor in a rat model related to the hypertension [58]. As reported in the literature, in an experimental rat model, the treatment with AA lead to a downexpression of NF*κ*B and a reduction of the excessive ROS production, responsible of blood vessel inflammation, as beneficial effects [59, 60]. p300 is a general transcriptional factor that can change the chromatin structure from heterochromatin to euchromatin, in order to enhance the binding of transcriptional factors to promoters [61]. p300 is necessary for the transcriptional activity of NF*κ*B, a crucial mediator of inflammatory responses [62, 63].

In our periodontitis in vitro model, LPS-G augmented the expression of NF*κ*B, MyD88, and p300. Moreover, our results reported a decrease expression of DNMT1 in LPS-G-treated samples. Meanwhile, the co-treatment of LPS-G and AA showed a downregulation of NF*κ*B, MyD88, and p300 and conversely an upregulation of DNMT1 in a similar way to the untreated cells.

Latest study has demonstrated that the NF*κ*B pathway can be initiated to promote proinflammatory cytokine expression in periodontitis [64]; in particular, NF*κ*B can be activated in hPDLSCs treated with LPS-G [65]. NF*κ*B signaling pathway is inhibited by miR-210, as reported by Zhang et al., leading to a reduction of the inflammatory cascade in osteoarthritis [66]. Our results suggest that the cells treated with LPS-G showed an activation of NF*κ*B signaling and a reduction in the expression of miR210, as also reported by Jia et al. [67]. miR-210 is decreased in patients with periodontitis in comparison to the healthy individuals. When cells were co-treated with LPS-G and AA, it showed an overexpression of miR210 that could inhibit the NF*κ*B pathway induced by AA treatment. LPS administration stimulated both p38 MAPK and NF*κ*B signaling pathways [67], while miR-210 overexpression repressed the p38MAPK/NF*κ*B pathway in LPS-stimulated PDLCs; similar results was obtained by Chen and Li [68].

## 5. Conclusions

In conclusion, the present work reported that miR-210 level was downregulated and the inflammatory signaling pathways are activated in an *in vitro* periodontitis model, while the co-treatment with AA could attenuate the inflammatory response. However, the results in our study should be further investigated by future *in vivo* models.

## Data Availability

The data used to support the findings of the present study are available from the corresponding authors upon request.
